# Barriers and facilitators to implementation of an exercise and education programme for osteoarthritis: a qualitative study using the consolidated framework for implementation research

**DOI:** 10.1007/s00296-024-05590-9

**Published:** 2024-04-22

**Authors:** Avantika Bhardwaj, Christine FitzGerald, Margaret Graham, Anne MacFarlane, Norelee Kennedy, Clodagh M. Toomey

**Affiliations:** 1 School of Allied Health, University of Limerick, Limerick, V94 T9PX Ireland; 2 Health Research Institute, University of Limerick, Limerick, V94 T9PX Ireland; 3 Department of Nursing & Midwifery, University of Limerick, Limerick, V94 T9PX Ireland; 4 School of Medicine, University of Limerick, Limerick, V94 T9PX Ireland; 5 Participatory Health Research Unit, University of Limerick, Limerick, V94 T9PX Ireland

**Keywords:** Barriers, Facilitators, Implementation, Exercise, OA, Self-management

## Abstract

Targeted efforts to better understand the barriers and facilitators of stakeholders and healthcare settings to implementation of exercise and education self-management programmes for osteoarthritis (OA) are needed. This study aimed to explore the barriers and facilitators to the implementation of Good Life with osteoArthritis in Denmark (GLA:D), a supervised group guideline-based OA programme, across Irish public and private healthcare settings. Interviews with 10 physiotherapists (PTs; 8 public) and 9 people with hip and knee OA (PwOA; 4 public) were coded by the Consolidated Framework for Implementation Research (CFIR) constructs in a case memo (summary, rationale, quotes). The strong positive/negative implementation determinants were identified collaboratively by rating the valence and strength of CFIR constructs on implementation. Across public and private settings, PTs and PwOA strongly perceived GLA:D Ireland as evidence-based, with easily accessible education and modifiable marketing/training materials that meet participants’ needs, improve skills/confidence and address exercise beliefs/expectations. Despite difficulties in scheduling sessions (e.g., work/caring responsibilities), PTs in public and private settings perceived advantages to implementation over current clinical practice (e.g., shortens waiting lists). Only PTs in public settings reported limited availability of internal/external funding, inappropriate space, marketing/training tools, and inadequate staffing. Across public and private settings, PwOA reported adaptability, appropriate space/equipment and coaching/supervision, autonomy, and social support as facilitators. Flexible training and tailored education for stakeholders and healthcare settings on guideline-based OA management may promote implementation. Additional support on organising (e.g., scheduling clinical time), planning (e.g., securing appropriate space, marketing/training tools), and funding (e.g., accessing dedicated internal/external grants) may strengthen implementation across public settings.

**Supplementary Information:**

The online version contains supplementary material available at 10.1007/s00296-024-05590-9.

## Introduction

Osteoarthritis (OA) is a major public health burden [1]. Several evidence-based guidelines for OA recommend exercise and education for self-management as first-line treatments [2, 3]. However, less than 40% of people with hip and knee OA (PwOA) receive guideline-based first-line treatments [4]. Global inequities and challenges within healthcare settings and service delivery, ageing populations, and higher obesity and physical inactivity rates may contribute to this evidence-to-practice gap [5–7]. Targeted efforts to strengthen the implementation of guideline-based healthcare services that may alleviate the burden of OA must be encouraged.

Good Life with osteoArthritis in Denmark (GLA:D) is a supervised group education and neuromuscular exercise self-management programme for OA [8]. Findings from 28,370 PwOA in the GLA:D data collection registry (Denmark, Canada, Australia) suggest that participation reduces pain intensity by 26–33% and improves quality of life by 12–26% [9]. To optimise healthcare service delivery for OA, GLA:D was cross-culturally adapted for implementation in Irish public and private healthcare settings using a participatory health research approach [10, 11]. In Ireland, a two-tiered healthcare system exists (closest to the UK and Australia), where cost and access to public services are associated with an individual’s circumstance (e.g., people with low incomes may be allocated medical cards granting access to some services for free) [12]. Approximately, 33% of the population have a medical card and 46% are covered by voluntary private health insurance, which may provide faster access to and better choice of providers/services [13]. Furthermore, rheumatologists are currently under-staffed in Ireland, with per capita numbers one of the lowest in Europe [14]. A better understanding of the multilevel factors of stakeholders and healthcare settings that influence the implementation of new healthcare services is needed to address the gap in guideline-based OA management.

Implementation science and the application of established theoretical frameworks consider multifactorial barriers and facilitators to programme implementation [15, 16]. Previously, the implementation of GLA:D Canada and GLA:D Australia were evaluated using mixed methods [17] and surveys [18], respectively. Implementation determinants, such as costs and training, were reported using the Reach, Effectiveness, Adoption, Implementation, and Maintenance (RE-AIM) framework. However, problems associated with applying RE-AIM have been highlighted regarding reporting of recruitment methods and/or costs and resources required [19]. The Consolidated Framework for Implementation Research (CFIR) is a comprehensive implementation science framework comprising of five domains: “Innovation”, “Outer Setting”, “Inner Setting”, “Individuals”, and “Implementation Process” [20]. Previously, CFIR has been used to identify the implementation barriers and facilitators of guideline-based programmes that may improve other healthcare services, including older adults with disabilities and frailty [21, 22]. CFIR’s inclusion of a broad range of implementation determinants may be useful across different healthcare systems, services, stakeholders, and societies globally. Thus, this study aims to use CFIR to qualitatively explore the barriers and facilitators to the implementation of a supervised group neuromuscular exercise and education self-management programme for OA (GLA:D Ireland) across public and private healthcare settings. The implementation determinants associated with a strong positive/negative influence on stakeholders and healthcare settings will be identified.

## Methods

### GLA:D Ireland

This qualitative study, as part of a larger study (IMPACT: IMPlementation of osteoArthritis Clinical guidelines Together), was conducted between 2022 and 2023. A convenience sample of PTs from different Irish healthcare settings was invited to participate in GLA:D training, with additional courses advertised nationally, across networks and clinical interest groups [10]. These PTs participated in a training course, between October 2021 and May 2022, developing essential skills and knowledge on guideline-based OA management and GLA:D Ireland protocol (e.g., delivering education, supervising neuromuscular exercise, and using the online data collection registry) [10]. After completion of the course, GLA:D-trained PTs used existing/new referral sources to invite PwOA (≥ 18 years old) to participate. Criteria excluding participation were the non-OA cause of symptoms (e.g., tumour), or other symptoms that are more pronounced than OA (e.g., fibromyalgia) [10]. GLA:D Ireland consisted of face-to-face supervised group education sessions (*n* = 2) and neuromuscular exercise sessions (*n* = 12 (twice a week)).

### Consolidated framework for implementation research

Implementation was assessed retrospectively, using CFIR as an evaluation framework. CFIR offers a structured approach developed from multiple implementation theories to promote effective implementation [20]. CFIR consists of 60 constructs sorted under five domains: “Innovation” “Outer Setting”, “Inner Setting”, “Individuals” and “Implementation Process”. Table 1 presents further details on the definitions and operalisation of CFIR domains.

**Table 1:** Operalisation of CFIR domains [41]

	Domain	Definition, according to CFIR authors	Operalisation
I	Innovation	The “thing” being implemented	GLA:D Ireland
II	Outer setting	The setting in which the inner setting exists	Irish healthcare setting (public or private)
III	Inner setting	The setting in which the innovation is implemented	Participant healthcare setting
IV	Individuals	The roles and characteristics of individuals involved in the innovation	PT and PwOA participating in GLA:D Ireland
V	Implementation process	The activities and strategies used to implement the innovation	Process of implementing GLA:D Ireland

*PT* physiotherapist, *PwOA* people with hip and knee OA, *GLA:D* Good Life with osteoArthritis in Denmark, *CFIR* consolidated framework for implementation research

### Sample

Inclusion criteria were (1) PTs who have implemented GLA:D Ireland and (2) PwOA who have taken part in GLA:D Ireland. Participants were contacted by email and maximum variation sampling was applied to include a balance of gender, affected joint, duration of pain, and healthcare settings to collect data from the widest range of implementation experiences. The sampling of new participants continued until data saturation.

### Data collection

A semi-structured interview guide including a range of open-ended questions/probes on participant experiences that were an implementation barrier and/or facilitator was developed using CFIR and prior literature [17] and is presented in Online Resource 1. The guide was tailored to gather specific information on GLA:D Ireland, with CFIR constructs informing operalisation. A pilot interview to test and refine the guide was undertaken. No revisions were necessary. Questions were related to programme delivery, logistics, fidelity, appropriateness and sustainability. Participant descriptions were probed to understand how implementation experiences related to each CFIR construct and the study aim. Interviews were conducted by a single investigator (AB) via a telephone call or online videoconferencing software Microsoft Teams (Microsoft Teams v.1.6, USA). AB is a PhD candidate evaluating the implementation of GLA:D Ireland, has trained in qualitative methodologies, and had no other interactions with participants. The interviews were audio-recorded and transcribed verbatim by AB, and personal identifiers were removed. Written consent was obtained from all participants prior to interviews. To minimise bias, AB reflexively assessed pre-existing perspectives using field notes to document decisions.

### Data analysis

Demographic characteristics were analysed quantitatively using frequency distributions. Interview data were interpreted by at least two investigators using a deductive content analysis approach first, appropriate for analysing data using an existing framework (CFIR) [23]. Any additional themes identified using an inductive approach were labelled as appropriate. Firstly, investigators (AB, CF, MG) familiarised themselves with the data through multiple readings of transcripts. Following this, transcripts were transferred to and managed with NVivo10 (QSR International). An NVivo10 template, pre-populated with CFIR construct definitions, was used [24] to develop codes for each transcript [25]. Based on these codes, case memos for each transcript including summary statements with quotations were developed and organised by each CFIR construct. This was a highly iterative process and involved refining CFIR constructs continuously within the scope of the study until the case memo for each transcript was complete (i.e., until data saturation). Next, each CFIR construct within each case memo was subjected to a rating process using a consensus approach [24, 25]. The ratings reflected the valence (positive/negative influence) and strength of each CFIR construct based on each case memo. CFIR constructs were rated as either: missing data (M), mixed positive and negative (X), strong negative (-2) or positive (+ 2), weak negative (-1) or positive (+ 1), or neutral/no influence on implementation (0). Table 2 presents further details on the criteria used to assign ratings. Once all CFIR constructs for all case memos were rated, we created a matrix that listed all the ratings using Microsoft Excel (Microsoft Excel v.16.59, USA). Once consensus on overall ratings for each CFIR construct was reached, we reviewed overall ratings to discern patterns that reflected participants’ descriptions. This allowed us to take a collaborative approach to identify implementation barriers and facilitators. During each step of the analysis, investigators continuously reviewed the codes and associated ratings to identify pre-existing biases or assumptions, to ensure they reflected participants’ descriptions of implementation barriers and facilitators, and to resolve any discrepancies.

**Table 2:** Criteria used to assign ratings to CFIR constructs in this study [25]

Rating	Criteria
– 2	A strong negative influence on the implementation of GLA:D Ireland. The participants provide several negative examples and use strong language to describe aspects of implementing GLA:D Ireland
– 1	A weak negative influence on the implementation of GLA:D Ireland. The participants provide a few negative examples and use weak language to describe aspects of implementing GLA:D Ireland
0	No influence on the implementation of GLA:D Ireland. The use of examples and strength of language are neutral, i.e., comments are related to a construct but have no bearing on the implementation
+ 1	A weak positive influence on the implementation of GLA:D Ireland. The participants provide few positive examples and use weak language to describe aspects of implementing GLA:D Ireland
+ 2	A strong positive influence on the implementation of GLA:D Ireland. The participants provide several positive examples and use strong language to describe aspects of implementing GLA:D Ireland
X	Neither positive nor negative influence on the implementation of GLA:D Ireland, i.e., comments are mixed or equally positive and negative
Missing	The participants did not provide any examples that may influence the implementation of GLA:D Ireland and/or comments were coded to another relevant construct

*CFIR* consolidated framework for implementation research, *GLA:D* good life with osteoArthritis in Denmark

## Results

### Sample

Interviews were conducted between 2022 and 2023 (i.e., within six months of having completed their respective GLA:D Ireland programmes). In total, 20 PTs and 35 PwOA were invited and of these, 10 PTs (50.0%) and 9 PwOA (25.7%) responded and agreed to be interviewed. The PT interviews ranged in duration from 29 to 54 min (telephone call *n* = 1), PwOA from 22 to 37 min (telephone call *n* = 9), and no repeat interviews were conducted. The majority of participants were female (PTs: 60.0%; *n* = 6 and PwOA: 77.8%; *n* = 7), living in the province of Connacht (PTs: 40.0%, *n* = 4) and Leinster (PwOA: 55.6%, *n* = 5), and reported public (PTs: 80.0%, *n* = 8) or private (PwOA: 55.6%, *n* = 5) healthcare settings. Detailed participant demographic characteristics can be found in Table 3.

**Table 3:** Demographic characteristics of the study sample

	PT demographics, *n* = 10	PwOA demographics, *n* = 9
*Age*:	*n* (%)	*n* (%)
50–59	–	2 (22.2)
60–69	–	5 (55.6)
70–79	–	2 (22.2)
*Gender*:		
Female	6 (60)	7 (77.8)
Male	4 (40)	2 (22.2)
*Province in Ireland***:**		
Munster	3 (30)	4 (44.4)
Ulster	2 (20)	–
Leinster	1 (10)	5 (55.6)
Connacht	4 (40)	–
*Healthcare setting*:		
Public	8 (80)	4 (44.4)
Private	2 (20)	5 (55.6)
*Duration of pain*:		
0 months–1 year	–	2 (22.2)
1–4 years	–	–
4–5 years	–	4 (44.4)
5–6 years	–	1 (11.1)
More than 10 years	–	2 (22.2)
*Most bothersome joint*:		
Knee	–	6 (66.7)
Hip	–	3 (33.3)

*PT* physiotherapist, *PwOA* people with hip and knee OA, – not applicable

### CFIR domains

Of the total 60 CFIR constructs, PTs identified 11 as barriers ( – 2 or  – 1), 33 as facilitators (+ 2 or + 1), 4 as both barriers and facilitators (X), 2 as neutral (0), and 10 as missing too much data to discern an influence on implementation (M). PwOA identified 2 CFIR constructs as barriers, 28 as facilitators, 2 as neutral, and 28 as missing. More facilitators were identified than barriers for both PwOA and PTs in the CFIR domains of “Innovation”, “Inner Setting”, “Individuals” and “Implementation Process”. However, more barriers were identified in the CFIR domain of “Outer Setting” for PTs. For a complete list of barriers and facilitators with associated ratings, see Online Resource 2.

Tables 4 and 5 list illustrative quotes for CFIR constructs with a (1) strong positive/negative and (2) mixed positive and negative influence on implementation for PTs and PwOA, respectively. PTs identified nine CFIR constructs as strong positive, 1 as strong negative, and 4 as mixed positive and negative influences on implementation. PwOA identified 15 CFIR constructs as strong positive influences on implementation. No CFIR constructs were identified by PwOA as strong negative or mixed positive and negative influences on implementation.

**Table 4:** CFIR construct ratings and PT barriers and facilitators to implementation of GLA:D

CFIR construct		Rating	Barrier or facilitator	Illustrative quote	
*Domain I: innovation*				Public healthcare setting	Private healthcare setting
Innovation evidence-base	–	+ 2	Facilitator	PT 3: Yeah, so really kind of I think the fact that GLA:D is so international at this stage, it’s become more and more popular. So many PTs talk about it on Twitter, it’s kind of become like more normal to kind of hear about it	PT 6: Yeah. Basically, with GLA:D you’re aware that maybe that’s the frontline evidence of handling hip and knee OA
Innovation relative advantage	–	+ 2	Facilitator	PT 1: And as I said to you prior to the course or during the training of the course, I was sceptical that it was just another exercise programme, which I guess it is just another exercise programme. But the way it’s based on the education around it, I think that education is powerful	PT 4: I feel it’s a much better model of care for some people to manage and empower them and give them a number of sessions that they can actually improve their strengths
Innovation design	–	+ 2	Facilitator	PT 9: Well, even though we give them and teach them exercises for hip and knee, a programme like this is structured, so it’s really good to have a structure and the evidence-based approach as well, because it has worked before and we know it will work, so that kind of helps	PT 4: The quality of the material is fantastic. It was really well laid out, there were pictures, good clarity in the text, very clearly shows their progressions and the booklet was excellent
*Domain II: outer setting*					
Financing	–	X	Barrier and facilitator	PT 5: Luckily, at the moment, the X have money for exercise, so they paid the rent on the hall, and they’re happy to do that for the second cohort. I don’t know if they’d be happy to do it because their view of it was that it was very much a pilot, which is fine	Missing
*Domain III: inner setting*					
Structural characteristics	Information technology infrastructure	X	Barrier and facilitator	PT 5: I wanted to deliver the exercise component in the community, but the site I’d chosen for the community didn’t have audio/visual facilities	Missing
	Work infrastructure	X	Barrier and facilitator	PT 1: So that’s a little bit difficult in a way that like that’s you know we’re only a small clinic here. We’ve only got 2 PTs. And so, the chances are someone is going to be off on annual leave	Missing
Tension for change	–	+ 2	Facilitator	PT 7: Previously, years ago, when treating patients with osteoarthritis, you’re going to keep getting them coming back to the door again and again	PT 4: We see people with subacute to chronic pain in the hip and knee joint, it relates to our strategy changes
Available resources	Funding	X	Barrier and facilitator	PT 3: I’m hoping to do two classes back-to-back, but I think in terms of kind of going forward long term, I definitely would look into kind of maybe getting funding for a bigger space	Missing
Access to knowledge & information	–	+ 2	Facilitator	PT 5: I thought the training was comprehensive. I thought it was excellent. I thought it covered everything that you need to know to get the programme off. I think it was kind of ideal, really, in terms of standardisation of the testing	PT 4: I thought there was a nice mix of lecture and practical. I thought the practical was really well delivered and it was good that we got the opportunity to practice on ourselves, practice with a partner. That was really good. Yeah, I thought the overall form of it was excellent. There was ample opportunity to ask questions, have some group discussions there learn a lot of people's questions.
*Domain IV: individuals*					
*Sub-Domain IV: characteristics*					
Need	–	+ 2	Facilitator	PT 2: Yeah, we all noticed functional improvements, and their day-to-day less pain with them walking downstairs, while they were at work. Some of them are caring for family members and found things like that got easier. Their strength improved and their stamina definitely	PT 4: It will certainly in some clients, develop their confidence. It will give them knowledge and ability to perform exercises on their own or following the class, the period of the class programme, and give them the opportunity to ask questions, therefore develop them, improve their knowledge on being realistic about failures and flareups and have been given them the knowledge to self-manage flareups as well
Capability	–	+ 2	Facilitator	PT 5: By week three, people are kind of into the swing of things and they know the exercises, they’re familiar with the exercises, they know how to modify	PT 6: Well, I suppose the main area is the ability to maybe progress the exercise and having a bit more confidence in that when they’re supervised, you’re a bit more, I was a bit more, sorry, comfortable, and confident in terms of progressing the exercises while watching the people complete them
Opportunity	–	– 2	Barrier	PT 10: Because obviously there’s a time commitment on our end as physios. And I suppose we need to make sure that we see enough patients in that time to make that investment worthwhile, if that makes sense	PT 6: If I didn’t have the clinic space I do here now, I’d probably dismiss it because there’s too much hassle involved in trying to find another space and then trying to factor in that on top of cost and time
Motivation	–	+ 2	Facilitator	PT 9: But when they are in a class, in a group, they are well able to when they see others doing the exercises. And once they know what level of pain is acceptable and if they’re able to continue with that, definitely, I can see after two weeks, they improve. And that kind of helps to keep up the motivation and keep up the exercises	PT 4: It is something that we will do more of depending on capacity. But if we get a new physio on board, it will mean increased time in the diary, I will deliver a second class in the week, a second group if needed, and that would be great for the whole population
*Domain V: implementation process*					
Engaging	Innovation deliverers	+ 2	Facilitator	PT 3: But certainly, it’s something whereby I think if there was a way I could kind of try and promote it a bit more. But I think word of mouth as well is helpful and I think definitely the more it becomes a thing, especially if we bring it kind of externally into the community, it’ll definitely kind of grow in popularity	PT 6: I probably have to do more of a push in terms of creating awareness about the GLA:D programme from the private practice side of it, just making GPs more aware of it and consultants

*PT* physiotherapist

**Table 5:** CFIR construct ratings and PwOA barriers and facilitators to implementation of GLA:D

CFIR construct			Rating	Barrier or facilitator	Illustrative quote
*Domain I: innovation*				Public healthcare setting	Private healthcare setting
Innovation evidence-base	–	+ 2	Facilitator	PwOA 9: Well, it was just so informative. It was just informative about how the body works, especially hips and knees and things like that, and as I say, just the whole mindset about dealing with pain and things like that	PwOA 6: Yeah, what I found them really useful to do before you actually start the programme because they gave some information in relation to the research that had been done, you know, up to date research. For example, I would have had some arthroscopies, I think they’re called, on my knee, where I would have gone to the doctor and I would have had issues with my knee, and I would have thought that he would have, in the past, I had scopes done on my knee. And the last time I went to the orthopaedic surgeon, I kind of thought, oh, I’m having issues. I thought he’d be able to go in and do another scope. But actually, when I did the education session and he said that the research is not there to support that and that was reiterated at the education sessions that scopes, that the evidence isn’t there that they’re useful
Innovation adaptability	–	+ 2	Facilitator	PwOA 7: Yeah, she explained. And that was another thing, of course. Like, you didn’t need equipment as such. You could use the wall. You could use chairs. You could use bottles of water for weights and things like that. Lastly, you didn’t have to go and buy special equipment, or you could use your stairs for the steps and things	PwOA 3: Yeah, no, I didn’t mind the group sessions. Like, people went off and did their own exercises, their own you’re in a room with people, we all have different speeds, and it was very well managed that way, and people weren’t expected to keep up with other people or anything like that, so that was grand
Innovation design	–	+ 2	Facilitator	PwOA 2: The delivery was first class. There was nothing that was left. There was nothing that was left to interpretation. Everything was quite thorough and very straightforward. The content was very informative, and the diagrams were particularly good	PwOA 5: Well, I mean, the information was given in a very clear and detailed way with diagrams and the whiteboards. It was all very clearly done. Absolutely no problem with that at all. It was very detailed. And he went over everything in great detail and took great care of it
*Domain II: outer setting*					
Local attitudes	–	+ 2	Facilitator	Missing	PwOA 4: How much I learned about PT because as an X-year-old who was addicted to exercise my whole life, had my regime, going to the gym, walking, you name it, I did it. And worked in X in the lab. So, I was always in touch with medical stuff and that. But PT was something, and no disrespect, I felt my daughter who studied PT, but just kind of went, well, I wouldn’t really need PT until now
*Domain III: inner setting*					
Structural characteristics	Physical infrastructure	+ 2	Facilitator	PwOA 9: Oh, definitely. It’s a big primary healthcare centre here we had in X. We’re very lucky. And there’s a whole floor of PT, which is brilliant. And they had all the equipment and the bars and the balls for doing other exercises. Everything was there	PwOA 4: It’s just fantastic. And I know we’re very privileged to have it with private patients, but my God, it’s just amazing. As an alternative to go, if you have an urgent issue, I’m not talking about heart and lungs or systemic, I’m talking about your joints or an injury or a wound. I just think it’s fantastic to have and it’s a beautiful premise
Culture	Recipient-centeredness	+ 2	Facilitator	PwOA 2: They actually helped you on and off with the bands and showed you how to use them and they gave each person that was on the course time and watched how they performed and corrected their movements if they needed to be corrected	PwOA 5: I mean, he was observing what we were doing and if we were doing it correctly in class, he reckoned we were able to do it at home. Just to pace yourself, really just don’t overdo it and stop or reduce the number of repetitions if you felt you were putting too much strain somewhere on something that might be a problem, particularly for you
Relative priority	–	+ 2	Facilitator	PwOA 2: Because one session led on to another and as the weeks went by, we increased the exercises and if you missed out on the session, then, it wasn’t really, you wouldn’t be as good going into the next session. You’d have to have resolved that you were going to turn up at every session	PwOA 8: I suppose the whole thing of having to go and do it every week and you had a kind of a target and a deadline and having to do be out there every week and get out there and you kind of felt good. I’m part of a little group here, I better get out and do my version not to miss out
Access to knowledge & information	–	+ 2	Facilitator	Missing	PwOA 4: Anything else? No, I have I mean, I have the handouts of the exercises, and I can always refer back to that
*Domain IV: individuals*					
*Sub-Domain IV: roles*					
Implementation leads	–	+ 2	Facilitator	PwOA 2: Well, we had two supervisors, there was the PT, and then there was another guy there who was an assistant, and they were fabulous	PwOA 3: No, it was all reasonably well presented by the X crowd
Sub-Domain IV: characteristics					
Need	–	+ 2	Facilitator	PwOA 7: Definitely, with regards to, my mobility is much better, and I can walk for longer without having this pain in my knee. So, I do spend longer, we say, when I go to town, I spend longer walking. It’s not an effort as such anymore	PwOA 3: And I could see the improvement in my knee strength, or in the muscles around the knee from week to week. And I saw the final session that we did where we did the measurements before and after. I could see a big improvement there
*Domain V: implementation process*					
Teaming		+ 2	Facilitator	Missing	PwOA 4: Because the other thing is the way you build up a rapport with those women in literally the few minutes before the class and after, possibly, you chat
Assessing context	–	+ 2	Facilitator	PwOA 7: It’s all explained to you when you start and there’s somebody there because it was nearly one on one, there’s somebody there all the time that you can ask or somebody to make sure your posture is right and things like that. Just like having a personal trainer as such	PwOA 6: Well, you obviously need to have it indoors, I think, so you need a sizable room. You need the props, like the big balls and chairs and other props
Engaging	Innovation deliverers	+ 2	Facilitator	PwOA 2: I would approach the consultants. The orthopaedic consultants, they have the people going into them and they don’t offer any other alternative except, well, I think you need a full knee replacement, I think you need a half knee replacement	Missing
	Innovation recipients	+ 2	Facilitator	PwOA 9: Recommend other people do, I would, I think we’re very lucky to get on the course, and I think it would help a lot of people, even if the classes were bigger	PwOA 5: Well, I think they should try it. There’s nothing to be lost by trying. It’s easy to access. It’s not a difficult programme. It’s something you can do at your own pace. You can increase the severity of it if you want to or if you’re able for it. I think it’s certainly worth exploring if you have issues with your joints
Reflecting & evaluating	Implementation	+ 2	Facilitator	Missing	PwOA 8: I think when we did the assessment, there was an assessment afterwards where you kind of did, the PT put you through the various, I think I did well in all those assessments. I think, apart from the hop thing, or you were to hop along, and I wasn’t able to do that. I kind of lost my balance a bit, but any of the other ones, I find my performance was better than when I did the initial assessment

*PwOA* people with hip and knee OA

### Innovation

“Innovation Evidence-Base” and “Innovation Design” were strong facilitators for all participants. PTs and PwOA believed that the programme was supported by peers/colleagues, up-to-date research, previous clinical/participant experience, and in line with current guidelines. Both reported that the programme marketing and training materials included high quality, effective resources that were designed to be easily accessible.

***PT 9:**** “The quality of the programme is really good and it’s well-structured and evidence-based.”*

***PwOA 6:**** “If you found a particular exercise didn’t suit you, they had an alternative one that you could do.”*

Only PTs reported “Innovation Relative Advantage” as a strong facilitator citing a clear advantage to implementing the programme. They perceived that the programme offers an alternative treatment/care pathway for PwOA, shortens waiting lists and encourages first-line treatment success.

***PT 10:**** “On average, these patients would be seen once a month for three months, whereas now you’re definitely getting a more kind of an intensive input granted over a shorter period of time.”*

Only PwOA reported “Innovation Adaptability” as a strong facilitator acknowledging that the programme materials and equipment were easily modifiable, and the structure could be tailored to fit participant needs.

***PwOA 7:**** “You didn’t have to go and buy special equipment; you could use your stairs for the steps.”*

### Outer setting

“Financing” was both a barrier and facilitator only for PTs. They perceived that while external funding (e.g., grants, reimbursements) is available to implement new healthcare services, access may be limited, and long-term sustainability is challenging.

***PT 1:**** “Maybe I do one in the morning somewhere and one in the afternoon somewhere else, but probably just won’t work out more because of funding than anything else.”*

Only PwOA reported “Local Attitudes” as a strong facilitator citing previous exercise beliefs/expectations and PTs encouragement to engage in the programme.

***PwOA 4:**** “Exercise has always been a huge part of my life.”*

### Inner setting

“Access to Knowledge and Information” was a strong facilitator for all participants. PTs and PwOA perceived that guidance and training to implement the programme was accessible, timely, and informative.

***PT 3:**** “The practical component of it and practicing on each other and practicing teaching it. Even though the exercises are simple, it’s just getting the confidence in doing them and getting a structure with doing them.”*

***PwOA 4:**** “And X always was great at sending YouTube videos so you could actually watch, because I’ve been doing them religiously and consistently, I know how to do them, but that was handy at the start, so that you could just go back to them to refresh your memory on how to do the exercise.”*

Only PTs found “Tension for Change” as a strong facilitator. They described first-hand experiences with PwOA that demonstrate a strong need for change in the healthcare setting.

***PT 2:**** “Always see so much osteoarthritis, especially hip and knee. I think it could definitely help with our waiting list.”*

“Information Technology and Work Infrastructure”, and “Available Funding” acted as both barriers and facilitators only for PTs. They found that while roles/responsibilities of individuals in the healthcare settings may be well-organised, technical systems, staffing levels, and lack of consistently available internal funding may not support day-to-day activities.

***PT 2:**** “Management had been pretty good, just good timing that they had funding to buy stuff.”*

Only PwOA reported “Physical Infrastructure”, “Recipient-Centredness”, and “Relative Priority” as strong facilitators. PwOA perceived that the features and layout of the healthcare setting supported functional performance as well as programme delivery. Additionally, they found that they received care that addressed their needs (e.g., supervision and autonomy). Further, supporting participation in the programme may be important.

***PwOA 9:**** “I made up my mind that this is a chance to help me, to help myself, and that’s the way I looked at it, and that’s what worked out”*

### Individuals: roles

Only PwOA found “Implementation Leads” as a strong facilitator. PwOA believed that the programme was well-supported by individuals with expert knowledge.

***PwOA 2:**** “The whole thing was conducted in a very professional manner.”*

### Individuals: characteristics

“Need” was a strong facilitator for PTs and PwOA. Both believed that the programme met the needs of participants including, functional improvements, pain reduction, increased strength and stamina and social engagement.

***PT 8:**** “From a social point of view, they did so well. From a PT point of view, they did very well. From the exercise point of view, they did very well. And from functionally in their activities of daily living, they did very well.”*

***PwOA 1:**** “I bought myself a step. So, I’ve been stepping on the step and stepping over the step.”*

“Capability” and “Motivation” were strong facilitators only for PTs. They found that participants were highly committed to their role in the programme, understood the exercise and education self-management strategies, and had high confidence in their skills and knowledge.

***PT 9:**** “I didn’t experience many challenges because I have done a lot of classes before.”*

Only PTs found “Opportunity” as a strong barrier. PTs perceived a lack of time and resources to implement the programme. Further, work/family commitments and the logistics of recruiting participants and scheduling appointments were reported as barriers.

***PT 5:**** “Time is a challenge because resources are finite and trying to establish the programme on top of my other role, which is in triage, I only have very limited PT time.”*

### Implementation process

“Engaging Innovation Deliverers” was a strong facilitator for PTs and PwOA. Participants believed that attracting participation from appropriate healthcare professionals (HCPs) is important for implementation. They described several activities that may promote engagement including education, training, research, data, social marketing and role modelling.

***PT 3: “****You could use the argument with managers that you are clearing this cohort off the list and providing them with a service, which is easy to continue.”*

***PwOA 2:**** “I think the first phone call would be the consultant.”*

“Teaming”, “Assessing Context”, “Engaging Innovation Recipients” and “Reflecting and Evaluating on the Implementation” were strong facilitators only for PwOA. They perceived that building a good support network with other participants was important for continued engagement. Also, the proximity of the class to their location, self-management education and supervision were positive influences on participation. Attracting participation from other PwOA to the programme is important, with participants suggesting word-of-mouth, role modelling, education and peer-to-peer rapport building strategies. Participants also believed that formal assessments to capture outcomes may improve programme implementation.

***PwOA 2:**** “If it was known in the different hospitals that do orthopaedic surgery to give people the option of participating in that, I think more people might take it up.”*

## Discussion

Consistent with previous research, our findings within the CFIR domains of “Innovation”, “Outer Setting”, “Inner Setting”, “Individuals” and “Implementation Process” highlighted how characteristics and preferences of stakeholders and healthcare settings affect implementation. New healthcare services that are guideline-based and supported by colleagues/management are positively associated with implementation [26]. Gleadhill et al. [27] found that integration was crucial, with 77% of PTs (*n* = 57) rating their colleague’s treatment choices as ‘important’ or ‘very important’. This indicates the importance of developing appropriate care pathways and partnerships between stakeholders and healthcare settings. Participant-reported recommendations to encourage participation included early access to training and knowledge on components and benefits of the programme, social/role modelling, peer-to-peer marketing/word-of-mouth, and targeted education on up-to-date guidelines. Likewise, Parmar et al. [26] found that developing multidisciplinary teams, brainstorming potential implementation challenges and solutions, assigning responsibilities, and promoting HCP buy-in are critical steps to “intentional implementation planning”. Moreover, collecting feedback on implementation outcomes as an indicator of programme effectiveness may be used to improve stakeholder buy-in [28].

The use of new healthcare services is further promoted if participants perceive that programme marketing and training materials are easily accessible, and modifiable to meet specific needs [29]. Our participants believed that the programme may be tailored to suit their needs citing examples such as flexibility to use alternative equipment and the option to refine exercises to fit the home/outdoor environment. Also, the development of alternative online-delivered exercise and education self-management programmes may alleviate limitations imposed by work/caring responsibilities, location, or symptoms [30]. Further, training on how to implement the programme, and perceived confidence in capabilities to effectively execute the required steps are important positive determinants [26, 31]. Similar to GLA:D trained PTs in Switzerland (*n* = 86) who reported limited time and lack of appropriate space in the healthcare setting [32], our participants emphasised the need for flexible training opportunities that may improve participation (e.g., fit clinical time, proximity of training centre). Interestingly, PwOA did not report a lack of appropriate space (physical/technical features) as an implementation barrier citing opposing experiences to PTs. Possible reasons for this contrast in our findings could be due to differences in participant healthcare settings; the majority of PwOA in our sample reported as “private” healthcare settings (55.6%, *n* = 5), whereas majority of PTs reported “public” (80.0%, *n* = 8). Ireland’s private system offers faster access to and better choice of providers/services for those who can afford voluntary health insurance or to pay out of pocket [12]. While the Irish Health Service Executive delivers direct and indirect services, access to public healthcare remains poor. Specifically, Irish PwOA are estimated to use healthcare services significantly more than those without OA, costing €13.6 million annually [33]. Notably, only PTs reported the availability of internal and external funding/grants or reimbursement models as important determinants of implementation success. They perceived that the availability of financial support to implement new healthcare services is limited, citing that long-term adoption of new programmes may be difficult. A systematic review of reviews also reported that the financial climate of healthcare settings (e.g., allocation of or grants from health authorities/government funds) affects the implementation of evidence-based guidelines and new responsibilities [34]. It is pertinent to note that PTs in private healthcare settings did not identify financial climate as an implementation barrier. This supports previous findings on inequities in healthcare financing between public and private healthcare settings [13, 35]. Due to such inequities, it is possible participants in private healthcare settings are less likely to have experienced setting-level barriers to guideline-based clinical practice. Advocating for improved access to grants and reimbursement models and the development of programme guidelines/toolkits for logistics and financing may be effective. Likewise, future implementation efforts may find it helpful to define the necessary components of exercise and education self-management programmes for OA allowing healthcare settings to adapt services as relevant to their contexts.

Also, participation in GLA:D Ireland was perceived to be more beneficial than current clinical practice. While the perceived relative advantage of new healthcare services is an important factor in implementation success [36], it may be further compounded by dissatisfaction with current clinical practices. For example, challenges within healthcare settings are a barrier to implementation and may promote relative priority for testing new guideline-based programmes that aid global efforts to strengthen healthcare settings [7]. Additionally, implementation success may be influenced by how well the programme meets the needs of participants and is aligned with their beliefs/expectations. HCPs who were strongly interested in prevention also perceived a chronic disease prevention and screening programme to be a good fit with their role [37]. Similarly, in the implementation evaluation study on GLA:D Australia, PTs (*n* = 1064) reported that addressing participant beliefs about OA and treatment options was key to improving programme reach [18]. Our participants also described how PTs and healthcare settings addressed their needs including, receiving coaching/supervision, treatment choice and low costs. In the cross-cultural adaptation study on GLA:D Canada, PTs (*n* = 58) also described operational processes that may be patient-centred including, flexibility to schedule sessions and encouraging self-efficacy [17]. Future strategies may focus on promoting PwOA exercise behaviours and providing self-management education for stakeholders that highlights the benefits and advantages of exercise and education self-management programmes for OA.

### Implications

Findings provide more specific knowledge on multilevel (programme, stakeholder and healthcare setting) implementation determinants of guideline-based healthcare services for OA. This comprehensive insight may guide research, policy, and practice towards the development of new healthcare strategies that are effective. Specifically, the identification of more strong implementation facilitators than barriers indicates that stakeholders and healthcare settings may acknowledge the importance of implementing guideline-based programmes for OA. It also suggests that future implementers must focus on addressing strong implementation barriers including, lack of time, unclear roles/responsibilities, limited internal and external financial support, and inappropriate space (physical and/or technical features). Further work on targeted strategies to address strong implementation determinants across varying geographical locations, healthcare settings, and stakeholders is needed. For example, a targeted strategy for a new healthcare service in the future may recommend an implementation checklist that requires implementers to develop multidisciplinary teams with designated responsibilities and/or to secure dedicated space/funding prior to the onset of the programme. Additionally, our findings on commonalities and differences between stakeholders (PTs vs PwOA) and healthcare settings (public vs private) may help provide evidence of the gap in the provision of guideline-based OA management. This is critical for supporting global priorities to shift healthcare policies and alleviate the OA burden. Specifically, future work must focus on advocating for equitable staffing levels, funding, and space to implement new guideline-based healthcare services. Also, our participants were offered GLA:D Ireland at no financial cost, and more work on the impact of limited public healthcare funding and out-of-pocket private healthcare costs on healthcare service utilisation is needed.

### Strengths and limitations

A strength of this study is its focus on the combination of theoretical framework-based and explorative approaches to interpret the experiences of PTs and PwOA. Using CFIR allowed for both a context-specific (rating each construct for each participant) and variable-oriented (rating each construct across all participants) approach to identifying implementation barriers and facilitators [38]. Additionally, using the updated CFIR framework based on user feedback [20] ensured that a broad range of outcomes were examined to identify what works where and why, and our definitions/approach to operationalisation may help establish and strengthen validated measures, relationships, and boundaries within CFIR. Further, reflexive assessment of reviewers’ beliefs/experiences helped counteract potential bias. The study sample was mostly female, older age, reported knee pain and were evenly located in public and private healthcare settings [39, 40]. Although reasons for non-response to interview requests were unclear, perhaps time constraints or lack of interest to continue participation may have contributed. While the robustness of the research approaches adds credence to the findings, at individual healthcare setting levels, the findings of this study must be contextualised in maximising generalisability.

## Conclusion

To alleviate the public health burden of OA, global efforts to strengthen healthcare service delivery must address implementation barriers and facilitators of new guideline-based healthcare services. This study qualitatively identified the implementation determinants of PTs and PwOA for a supervised group neuromuscular exercise and education self-management programme for OA in public and private healthcare settings. Implementation may be strengthened by tailored training and education for stakeholders on the components and benefits of the programme, with more guidance needed for public healthcare settings on organising (e.g., scheduling clinic time, assigning responsibilities), planning (e.g., securing appropriate space, marketing/training tools), and funding (e.g., accessing dedicated internal/external grants). Future healthcare policy and practice may benefit from a framework-based approach to evaluating implementation determinants that may be applicable across broad contexts. Such contextualised research-driven strategies may support the future development of guideline-based healthcare services that encourage access to high-quality, equitable healthcare.

### Supplementary Information

Below is the link to the electronic supplementary material.

Supplementary file1 (DOCX 26 KB)

Supplementary file2 (DOCX 26 KB)

## Data Availability

The dataset supporting the conclusions of this article is available under the terms of the Creative Commons Attribution 4.0 International (CC BY 4.0) from Zenodo at: 10.5281/zenodo.10473852.

## Declarations

### Congress abstract publication

Data from the current study will be presented at one scientific meeting. Bhardwaj, A., FitzGerald, C., Graham, M., MacFarlane, A., Kennedy, N. and Toomey, CM. (in press). ‘Barriers and Facilitators to Implementation of an Exercise and Education Programme for Osteoarthritis: A Qualitative Study Using the Consolidated Framework for Implementation Research.’ Osteoarthr Cartil.
