# Supplementary material for: Barriers and facilitators to implementation of an exercise and education programme for osteoarthritis: a qualitative study using the consolidated framework for implementation research
Source: Rheumatol Int. 2024 Apr 22;44(6):1035–50. doi: 10.1007/s00296-024-05590-9 (PMC11108926; doi:10.1007/s00296-024-05590-9)
Supplement: Supplementary file 1 — Supplementary file1 (DOCX 26 KB) [file 296_2024_5590_MOESM1_ESM.docx]

Barriers and Facilitators to Implementation of an Exercise and Education Programme for Osteoarthritis: A Qualitative Study Using the Consolidated Framework for Implementation Research

Journal name

Rheumatology International

Author names

Avantika Bhardwaj^1,2^, Christine FitzGerald^1,2^, Margaret Graham^2,3^, Anne MacFarlane^4,5^, Norelee Kennedy^1,2^, Clodagh M. Toomey^1,2,5^

Corresponding author

Avantika Bhardwaj ([avantika.bhardwaj@ul.ie](mailto:avantika.bhardwaj@ul.ie)); School of Allied Health, University of Limerick, Limerick, V94 T9PX, Ireland. ORCID ID: 0000-0002-9482-5884.

**Appendix A.** Semi-structured interview guide for physiotherapists (PTs) and people with hip and knee OA (PwOA).

**Semi-structured interview guide for PT interviews**

1. DELIVERY: How did you deliver the programme? (i.e., face-to-face, online or combination)
2. OVERALL EXPERIENCE/BARRIERS: Please tell me about your experiences providing the education and exercise programme to people with hip/knee OA.

Probes: (cover exercise, education, supervision, group element, face to face or online)

- What did you like about the programme?
- What worked well?
- How well do you think the intervention will meet the needs of the individuals served by your organization?
- Were you satisfied with the programme overall?
- What challenges were encountered providing the programme?
- What would be your advice to others to avoid identified challenges?

Additional online probes:

- How do you think it compares to consulting with patients face to face?
- How did the online nature of the programme affect your communication style and the methods you use to develop a relationship with your patients?

1. FACILITATORS: Is there anything you wish you had known that would have helped you in delivering the programme?

Probes:

- How did you find the training you received to deliver the programme?
- How did you find the process of organizing programme delivery?
- How did you find the process of entering patient data on the patient database?
- Were there any additional knowledge or supports needed to provide the patient education?
- Were there any additional knowledge or supports to provide the patient exercise component?

Additional online probes:

- Were there any additional knowledge or resources to support online delivery?

1. LOGISTICS AND FIDELITY: If other therapists were going to start providing the programme, what advice would you give them and/or the clinic manager?

Probes:

- What is your perception of the quality of the supporting materials, packaging, and bundling of the intervention for implementation?
- What suggestions or recommendations would you offer related to educating and preparing the therapists to deliver the programme?
- What suggestions or recommendation would you provide concerning the logistics of programme delivery for the facility?
- Were there specific resource and cost implications?
- Did you make specific changes to the original programme? What changes did you make and why?

1. APPROPRIATENESS AND SUSTAINABILITY – Do you intend to continue delivering the programme?

Probes:

- How compatible is the GLA:D programme in your setting?
- What kind of supporting evidence or proof is needed about the effectiveness of the intervention to get staff on board?
- Does the programme help to address a particular issue or problem that was there?
- How complicated is the intervention? Please consider the following aspects of the intervention: duration, scope, intricacy, and number of steps involved and whether the intervention reflects a clear departure from previous practices.
- How does the intervention compare to other similar existing programmes in your setting?
- To what extent would implementing the intervention provide an advantage for your organization compared to other organizations in your area?
- Is there a competitive advantage?
- Is there something about the intervention that would bring more individuals into your organization, instead of another one in your area?
- What would you need to help you to continue delivering the programme long term?
- What kind of financial or other incentives influenced the decision to implement the intervention?

Additional online probes:

- What would your preference be for delivery (face to face or online)? Why?
- What advantages or disadvantages do you see that the online offers over in-person visits?
- How well do you think your patients understood the exercises and physical activity plan you prescribed?
- How confident were you that your patients could perform the exercises safely and effectively at home on their own?
- How confident were you that your patients would adhere to the exercise/activity programmes?

1. Is there anything else you’d like to tell me about the programme and its delivery?

**Semi-structured interview guide for PwOA interviews**

1. DELIVERY: How did you receive the programme? (i.e., face-to-face, online or combination)
2. OVERALL EXPERIENCE/BARRIERS: Please tell me about your experiences in taking part in the GLA:D Ireland education and exercise programme.

Probes: (cover exercise, education, timing, supervision, group element, face to face or online, any changes in symptoms, surveys, any costs, or accessibility issues)

- What did you like about the programme?
- What worked well?
- Were you satisfied with the programme overall?
- What challenges were encountered taking part the programme?
- What would be your advice to others to avoid identified challenges?

Additional online probes:

- How do you think it compares to consulting with physiotherapists face to face?
- How did the online nature of the programme affect your communication style and how you developed a relationship with your physiotherapists?
- Did you encounter any problems with setup for online classes (software, internet, space, equipment)? Did you need/get any extra assistance from your physiotherapist or family/friends?

1. FACILITATORS: Is there anything you wish you had known at the beginning of the programme that you did not know about?

Probes:

- Participant information sheets and information from your physiotherapist?
- Time commitment to attend sessions and complete surveys?
- Additional knowledge or supports for patient education?
- Additional knowledge or supports for patient exercise component?

Additional online probes:

- Were there any other supports that would have helped with the online programme?

1. APPROPRIATENESS AND SUSTAINABILITY – Do you intend to continue doing the exercises?

Probes:

- Do you use the information that you have learned from the programme currently or plan to?
- Are you confident performing the exercises in your own home?
- Did the programme address a particular issue or problem that you had?
- Do you think you have increased your physical activity and are there any particular resources you have used to do this? (e.g., joined a walking group, sports club)
- Would you recommend this programme to other people with osteoarthritis and if so, what advice would you give them?

Additional online probes:

- How well do you think you understood the exercises and physical activity plan you were prescribed?
- How confident were you that you could perform the exercises safely and effectively at home on your own?
- How confident were you that you would adhere/stick to the exercise/activity programmes?
- What would your preference be for delivery (face to face or online)? Why?
- What advantages or disadvantages do you see that the online offers over in-person visits?
- What advice would you give to people who are unsure about doing the programme online?
- Is there anything else you’d like to tell me about the programme and its delivery?
