# Supplementary material for: Barriers and facilitators to implementation of an exercise and education programme for osteoarthritis: a qualitative study using the consolidated framework for implementation research
Source: Rheumatol Int. 2024 Apr 22;44(6):1035–50. doi: 10.1007/s00296-024-05590-9 (PMC11108926; doi:10.1007/s00296-024-05590-9)
Supplement: Supplementary file 2 — Supplementary file2 (DOCX 26 KB) [file 296_2024_5590_MOESM2_ESM.docx]

Barriers and Facilitators to Implementation of an Exercise and Education Programme for Osteoarthritis: A Qualitative Study Using the Consolidated Framework for Implementation Research

Journal name

Rheumatology International

Author names

Avantika Bhardwaj^1,2^, Christine FitzGerald^1,2^, Margaret Graham^2,3^, Anne MacFarlane^4,5^, Norelee Kennedy^1,2^, Clodagh M. Toomey^1,2,5^

Corresponding author

Avantika Bhardwaj ([avantika.bhardwaj@ul.ie](mailto:avantika.bhardwaj@ul.ie)); School of Allied Health, University of Limerick, Limerick, V94 T9PX, Ireland. ORCID ID: 0000-0002-9482-5884.

**Appendix B.** CFIR construct ratings and barriers and facilitators to implementation of GLA:D.

|  | Construct | Sub-construct | Rating, influence on implementation | | Barrier or facilitator to implementation | |
| --- | --- | --- | --- | --- | --- | --- |
|  | **Domain I: Innovation** | | **PT** | **PwOA** | **PT** | **PwOA** |
| 1 | Innovation Source | - | M | M | M | M |
| 2 | Innovation Evidence-Base | - | +2 | +2 | Facilitator | Facilitator |
| 3 | Innovation Relative Advantage | - | +2 | +1 | Facilitator | Facilitator |
| 4 | Innovation Adaptability | - | +1 | +2 | Facilitator | Facilitator |
| 5 | Innovation Trialability | - | +1 | M | Facilitator | M |
| 6 | Innovation Complexity | - | +1 | +1 | Facilitator | Facilitator |
| 7 | Innovation Design | - | +2 | +2 | Facilitator | Facilitator |
| 8 | Innovation Cost | - | -1 | +1 | Barrier | Facilitator |
|  | **Domain II: Outer Setting** | | **PT** | **PwOA** |  |  |
| 9 | Critical Incidents | - | -1 | -1 | Barrier | Barrier |
| 10 | Local Attitudes | - | -1 | +2 | Barrier | Facilitator |
| 11 | Local Conditions | - | -1 | M | Barrier | M |
| 12 | Partnerships & Connections | - | -1 | M | Barrier | M |
| 13 | Policies & Laws | - | +1 | M | Facilitator | M |
| 14 | Financing | - | X | M | Barrier and facilitator | M |
| 15 | External Pressure | Societal Pressure | M | M | M | M |
| 16 |  | Market Pressure | +1 | M | Facilitator | M |
| 17 |  | Performance-Measurement Pressure | +1 | M | Facilitator | M |
|  | **Domain III: Inner Setting** | | **PT** | **PwOA** |  |  |
| 18 | Structural Characteristics | Physical Infrastructure | -1 | +2 | Barrier | Facilitator |
| 19 |  | Information Technology Infrastructure | X | M | Barrier and facilitator | M |
| 20 |  | Work Infrastructure | X | M | Barrier and facilitator | M |
| 21 | Relational Connections | - | -1 | M | Barrier | M |
| 22 | Communications | - | M | M | M | M |
| 23 | Culture | Human Equality-Centeredness | M | M | M | M |
| 24 |  | Recipient-Centeredness | +1 | +2 | Facilitator | Facilitator |
| 25 |  | Deliverer-Centeredness | +1 | M | Facilitator | M |
| 26 |  | Learning-Centeredness | +1 | 0 | Facilitator | Neutral |
| 27 | Tension for Change | - | +2 | +1 | Facilitator | Facilitator |
| 28 | Compatibility | - | -1 | M | Barrier | M |
| 29 | Relative Priority | - | +1 | +2 | Facilitator | Facilitator |
| 30 | Incentive Systems | - | +1 | M | Facilitator | M |
| 31 | Mission Alignment | - | M | M | M | M |
| 32 | Available Resources | Funding | X | M | Barrier and facilitator | M |
| 33 |  | Space | -1 | M | Barrier | M |
| 34 |  | Materials & Equipment | +1 | +1 | Facilitator | Facilitator |
| 35 | Access to Knowledge & Information | - | +2 | +2 | Facilitator | Facilitator |
|  | **Domain IV: Individuals** | |  |  |  |  |
|  | **Sub-Domain IV: Roles** | | **PT** | **PwOA** |  |  |
| 36 | High-level Leaders | - | +1 | +1 | Facilitator | Facilitator |
| 37 | Mid-level Leaders | - | M | M | M | M |
| 38 | Opinion Leaders | - | M | M | M | M |
| 39 | Implementation Facilitators | - | +1 | M | Facilitator | M |
| 40 | Implementation Leads | - | M | +2 | M | Facilitator |
| 41 | Implementation Team Members | - | +1 | M | Facilitator | M |
| 42 | Other Implementation Support | - | 0 | +1 | Neutral | Facilitator |
| 43 | Innovation Deliverers | - | M | M | M | M |
| 44 | Innovation Recipients | - | M | M | M | M |
|  | **Sub-Domain IV: Characteristics** | | **PT** | **PwOA** |  |  |
| 45 | Need | - | +2 | +2 | Facilitator | Facilitator |
| 46 | Capability | - | +2 | +1 | Facilitator | Facilitator |
| 47 | Opportunity | - | -2 | +1 | Barrier | Facilitator |
| 48 | Motivation | - | +2 | +1 | Facilitator | Facilitator |
|  | **Domain V: Implementation Process** | | **PT** | **PwOA** |  |  |
| 49 | Teaming |  | +1 | +2 | Facilitator | Facilitator |
| 50 | Assessing Needs | Innovation Deliverers | +1 | M | Facilitator | M |
| 51 |  | Innovation Recipients | +1 | +1 | Facilitator | Facilitator |
| 52 | Assessing Context | - | +1 | +2 | Facilitator | Facilitator |
| 53 | Planning | - | +1 | M | Facilitator | M |
| 54 | Tailoring Strategies | - | +1 | +1 | Facilitator | Facilitator |
| 55 | Engaging | Innovation Deliverers | +2 | +2 | Facilitator | Facilitator |
| 56 |  | Innovation Recipients | +1 | +2 | Facilitator | Facilitator |
| 57 | Doing | - | 0 | 0 | Neutral | Neutral |
| 58 | Reflecting & Evaluating | Implementation | +1 | +2 | Facilitator | Facilitator |
| 59 |  | Innovation | +1 | +1 | Facilitator | Facilitator |
| 60 | Adapting | - | -1 | -1 | Barrier | Barrier |

Note: PT: physiotherapist; PwOA: people with hip and knee OA; M: missing.
